# Progress in Laryngology

**Published:** 1900-09-15

**Authors:** 


### PROGRESS IN LARYNGOLOGY.

KYLE[1] deals with seven varieties of nasal deflections: (1) The split cartilaginous septum with bulging into both nostrils; (2) deviation of the columnar cartilage; (3) simple deflection in which the cartilage is very thin; (4) the letter S deflection; (5) deflection of the cartilage with involvement of the bony septum; (6) deflection due to the splitting of the cartilage with bulging on one side only; (7) deflection in which there is redundancy of tissue overlapping the septum, and extending close to the floor of the nose. The causes of deflections he divides into those from disease, those from trauma, and congenital deflections. The disease may occur directly in the structure or as a secondary condition depending upon some constitutional lesion. Purulent rhinitis and struma, and rickets may weaken the septum, so as to allow of slight deflection. The muscular action of the external nasal muscles may also have an effect upon an inflamed septum. Diseases of the teeth, especially during first dentition, may also bring about deviation. Superficial ulcerations, perichondritis, enlarged turbinated bones, the uric acid diathesis with perichondritis, all tend to deflection. The deviation from injury generally occurs in childhood, but as a rule it is not recognised until adult life. To cause deflection the blow must be sufficient to involve the bony septum. A frequent deflection of traumatic origin is one which is due to a dislocation of the anterior end of the septum from the columnar cartilage. This is found just within the anterior nares extending to the muco-cutaneous surface. Kyle believes that many of the so-called congenital deformities are due to the pressure upon the soft cartilaginous bones of the nose during labour. He lays great stress upon necessity of a free passage of air through the nostril for the regular development of the nasal fossæ and symmetry of the facial bones. The "worst feature of these developmental deformities is that unless perfect nasal respiration is established early in life—*i.e.*, before the fifth or sixth year, or not later than the seventh—the bony and cartilaginous framework becomes so firm that little can be done toward increasing the nasal space for breathing, and the individual will of necessity be a mouth-breather for life." He recommends as treatment in simple cases flexible or malleable tubes, made at first to fit the deflection, and afterwards gradually widened according to the amount of pressure required. If, however, the deflection cannot be bent back, surgical interference is necessary. The operation recommended by Kyle, if the curve extends down to the floor of the nose, is to make an incision on the opposite side of the deflection close to the base, and through the mucous membrane to the cartilage; by means of a saw the cartilage is then divided to one-third of its thickness: the whole is then moulded into position by forceps, and retained by means of tubes similar to those mentioned above. If the bony portion be involved it is necessary to saw at two points, so as to divide the septum into equal thirds. If the deflection is vertical, the incisions must be vertical. Should enlarged turbinated bodies be present they should be removed before straightening the septum. In cases where there is a great redundancy of tissue, the mucous membrane should be dissected up, and a V-shaped piece of cartilage should be removed. Spurs may also be treated in this manner.

Bosworth,[2] in operating upon nasal deflections, finds the saws named after himself and cocaine anæsthesia all that is necessary. "The operation is done at one office sitting, and the new straight septum is sawn out of the old crumpled one, much as a straight board is sawn out of a crumpled log." Asch makes a cross-cut through the extremity of the deflection by means of two pairs of scissors, and the flaps are pushed through into the opposite nostril and retained there by perforated vulcanite plugs. Roe, Beaman Douglas, and Gleeson have methods of their own, but in all a perforation of the cartilage is made, and the flap pushed into the opposite nostril. The perforation frequently remains, and gives rise to some inconvenience.

De Blois[3] considers that "broken" noses, so-called, are not as a rule really fractured, but rather cases of bony displacement and dislocation. There might be a dislocation of the nasal bone, or fracture of the nasal process of the superior maxilla, or of the zygoma. Injuries might result during parturition, or in infancy from the constant impact of the nose in nursing, or sleeping against the mammæ or pillow. The "upper cut" blow of the boxers gives rise to injury of the septum, with detachment from the subjacent parts. The "side" blow, a double dislocation of the nasal bones; whilst the direct "front" blow drives the internal nasal bones downward and outward. For treatment De Blois recommends reduction of the dislocation, followed by proper manipulation, which dispenses with external apparatus. As an internal splint he recommends a stiff rubber tube; for external, plaster of Paris bandages. Mayer prefers a gutta-percha splint, which can be moulded to each individual case. Roe commends a thin metal splint externally with internal dressing, adhesive plaster being used as a retaining material. Swain thinks that if the two nasal bones were brought into proper approximation they would support each other. He would dispense with all apparatus if the patient could be seen frequently. Simpson advocates Bernay's sponges. Chappell uses the sponges attached by iodoform collodion to a thin plate of gutta-percha.

[1] Laryngoscope, Jan., 1900 [2] Quarterly Med. Jour., May, 1900. [3] Med. Rec., May 5, 1900.

